# Both Mother and Infant Require a Vitamin D Supplement to Ensure That Infants’ Vitamin D Status Meets Current Guidelines

**DOI:** 10.3390/nu10040429

**Published:** 2018-03-29

**Authors:** Fariba Aghajafari, Catherine J. Field, Amy R. Weinberg, Nicole Letourneau, APrON Study Team

**Affiliations:** 1 Departments of Family Medicine and Community Health Sciences, Cumming School of Medicine, University of Calgary, Calgary, AB T2N 1N4, Canada; 2 Department of Agricultural, Food and Nutritional Science, University of Alberta, Edmonton, AB T6G 2R3, Canada; cjfield@ualberta.ca (C.J.F.); aweinber@ualberta.ca (A.R.W.); 3 Faculty of Nursing and Cumming School of Medicine, University of Calgary, Calgary, AB T2N 1N4, Canada; nicole.letourneau@uclagary.ca; 4 Department of Pediatrics, Cumming School of Medicine, University of Calgary, Calgary, AB T2N 1N4, Canada

**Keywords:** vitamin D, 25(OH)D, breastfeeding, pregnancy, infant

## Abstract

We examined the association between maternal vitamin D intake during breastfeeding with their infants’ vitamin D status in infants who did or did not receive vitamin D supplements to determine whether infant supplementation was sufficient. Using plasma from a subset of breastfed infants in the APrON (Alberta Pregnant Outcomes and Nutrition) cohort, vitamin D status was measured by liquid chromatography-tandem mass spectrometry. Maternal and infants’ dietary data were obtained from APrON’s dietary questionnaires. The median maternal vitamin D intake was 665 International Units (IU)/day, while 25% reported intakes below the recommended 400 IU/day. Of the 224 infants in the cohort, 72% were exclusively breastfed, and 90% were receiving vitamin D supplements. Infants’ median 25(OH)D was 96.0 nmol/L (interquartile ranges (IQR) 77.6–116.2), and 25% had 25(OH)D < 75 nmol/L. An adjusted linear regression model showed that, with a 100 IU increase in maternal vitamin D intake, infants’ 25(OH)D increased by 0.9 nmol/L controlling for race, season, mid-pregnancy maternal 25(OH)D, birthweight, and whether the infant received daily vitamin D supplement (β = 0.008, 95% confidence interval (CI) 0.002, 0.13). These results suggest that, to ensure infant optimal vitamin D status, not only do infants require a supplement, but women also need to meet current recommended vitamin D intake during breastfeeding.

## 1. Introduction

Exclusively breastfed infants who are not supplemented with vitamin D are at increased risk for insufficient vitamin D and associated short- and long-term bone health consequences [1,2,3]. Additionally, low vitamin D status has been associated with increased maternofetal-related morbidity, including preeclampsia, gestational diabetes, delivery of small for gestational age infants, and juvenile onset type 1 diabetes [4,5,6]. The vitamin D content of breast milk is very much dependent on maternal vitamin D status [7,8,9]. Health Canada [10] and the Institute of Medicine (IOM) [11] have recommended 600 International Units (IU)/day of vitamin D for pregnant and breastfeeding women and 400 IU/day for infants, with the goal of achieving sufficient vitamin D status in the infant. However, studies in pregnant and breastfeeding mothers have shown a large proportion of these women having suboptimal vitamin D status despite reporting intakes that meet these recommendations [12,13,14,15,16].

Optimal vitamin D intake, as well as optimal 25(OH)D concentration, for breastfeeding mothers and their infants is not fully established [11,17,18]. The IOM has recommended a 25(OH)D cut-point of >50 nmol/L to define vitamin D sufficiency [11], but some studies suggest that an optimal level for bone health might be somewhat higher [19,20,21,22]. Considering these studies, Osteoporosis Canada [17] and the Endocrine Society [18] have recommended a serum concentration of >75 nmol/L as the target for optimal bone health.

Clinical trials of vitamin D supplementation during breastfeeding have shown that an intake of 400 IU/day by breastfeeding mothers was not enough to achieve a vitamin D status >75 nmol/L in their infants [23,24,25]. There is data to suggest maternal daily doses of 4000–6400 IU/day are needed to improve and maintain 25(OH)D concentration ≥75 nmol/L in exclusively breastfed infants who are not taking any vitamin D supplements [23,24,25]. Consistent with this, a study examining different levels of vitamin D supplementation on vitamin D status in healthy breastfed infants showed supplementing infants with 400 IU/day achieved a 25(OH)D concentration ≥75 nmol/L in only 55% of infants [26]. Intakes of up to 1000 IU/day by infants and 4000–10,000 IU/day by adults, including breastfeeding mothers, is recognized as intakes without negative health consequences (UL, Tolerable Upper Intake Level) by the IOM [11].

Although the literature on vitamin D in breastfeeding mothers and infants is growing, most of the recently published studies of breastfeeding mothers have focused on either mothers or infants as a method of improving/maintaining vitamin D status in infants [23,24,25,26]. As current recommendations are aimed at both mother and infant, it is logical and important to consider both.

We examined the association between maternal vitamin D intake during breastfeeding with their infants’ vitamin D status in infants who did or did not receive vitamin D supplements to determine whether infant supplementation was sufficient.

## 2. Materials and Methods 

This study is a secondary analysis of the APrON study (Alberta Pregnancy Outcomes and Nutrition; http://www.apronstudy.ca/), a prospective cohort of pregnant women and their children residing in Calgary and Edmonton (Alberta, Canada). APrON recruited a total of 2140 women between March 2009 and July 2012; 500 blood samples were available from their infants at three months of age. All participants provided informed consent to permit use of their data and blood samples for various research questions. The University of Calgary Health Research Ethics Board and the University of Alberta Health Research Ethics Biomedical Panel approved the project. Full details of the APrON study are described elsewhere [27].

### 2.1. Dietary Data

At each pregnancy visit and at 3 months postpartum, women were asked to describe in detail the quantity and type of foods and beverages consumed in the previous 24-h period (midnight to midnight). In addition, a Supplemental Intake Questionnaire (SIQ) [28] was developed for the APrON study that was based on questionnaires used in previous studies [28], and was adapted for use with pregnant women. This questionnaire was designed to capture the daily estimated intake of natural health products during each trimester of pregnancy [28]. Furthermore, mothers answered questions at 3, 6, 12, 24, and 36 months postpartum about infant feeding practices, including whether they were providing a daily vitamin D supplement to their infant [27].

### 2.2. Biological Samples

Maternal blood sample was collected at each clinic visit by a certified phlebotomist. In addition, an infant blood sample was collected at 3 months of age by a certified phlebotomist. Plasma was separated immediately, aliquoted, and stored in micro-centrifuge tubes at −80 °C for future analysis [27]. All frozen collected plasma was assayed alongside plasma quality control samples and standard reference materials using a clinically validated liquid chromatography tandem mass spectrometry (LC-MS/MS) assay at the laboratory of Doctor’s Data Inc. (St. Charles, IL, USA) [29], which measured 25(OH)D_2_, 25(OH)D_3_, and 3-epi-25(OH)D_3_ and demonstrated an intra-assay coefficient of variability of 4.6% at 28.3 nmol/L, 3.3% at 83.9 nmol/L, and 3.9% at 5.8 nmol/L for 25(OH)D_2_, 25(OH)D_3_, and 3-epi-25(OH)D_3_, respectively. Information on processing blood samples and integrity of plasma samples has been previously reported [29]. The concentrations of 25(OH)D_2_, 25(OH)D_3_, and 3-epi-25(OH)D_3_ in plasma were determined using LC-MS/MS (the National Institute of Standard and Technology (NIST) standards and Vitamin D External Quality Assessment Scheme (DEQAS) procedures were followed), as previously described [29].

### 2.3. Statistical Analysis

Results are presented for plasma 25(OH)D_2_, 25(OH)D_3_, and 3-epi-25(OH)D_3_ as means and standard deviations (SD) and medians and interquartile ranges (IQR). The prevalence rates of vitamin D deficiency and insufficiency in infants were estimated as percentages. Plasma concentrations of 25(OH)D at <25 nmol/L, <50 nmol/L, and <75 nmol/L [11,17,18] were used to reflect several laboratory definitions of vitamin D status currently used. The correlation between maternal vitamin D intake and infants’ 25(OH)D concentration was determined using the Pearson correlation coefficient test. A multiple linear regression analysis was used to examine the association between infants’ plasma 25(OH)D (D_2_ + D_3_) concentration and mothers’ estimated dietary vitamin D intake (supplements and diet) adjusted for the potential covariates of birthweight, second trimester (mid-pregnancy) maternal plasma 25(OH)D (D_2_ + D_3_), season (summer from 1 May–31 October and winter from 1 November–30 April), race (Caucasian vs. non-Caucasian) and infants’ vitamin D supplement status (yes or no, as the actual dose was not measured). All analyses were conducted using SPSS (V22.0, IBM Corporation, Armonk, NY, USA). *p*-values < 0.05 were considered statistically significant.

## 3. Results

We analyzed 224 blood samples of infants whose mothers’ vitamin D status (at second trimester) and intake was available (Table 1). Fifty-five percent of infants were male, with a mean birthweight of 3377.8 ± 478.3 g. Data on infant feeding was accessible for 209 infants for whom vitamin D status was available; 72% were exclusively breastfed, 12% were formula fed, and 16% were mix fed (receiving both breast milk and formula). Ninety percent of exclusively breastfed infants were provided a vitamin D supplement. Overall, 80% of mothers in the APrON study provided vitamin D supplementation to their infants [30].

25(OH)D_2_, 25(OH)D_3_, 3-epi-25(OH)D_3_ were identified in 224 plasma samples at three months of age, and 3-epi-25(OH)D_3_ contributed 15% of the total vitamin D status (Table 2). Infants’ median (25(OH)D_2_ + 25(OH)D_3_) was 96.0 (IQR 77.6–116.2). Twenty-three percent of infants had 25(OH)D < 75 nmol/L, and 8% had 25(OH)D < 50 nmol/L. These percentages were slightly higher in exclusively breastfed infants (25% < 75 nmol/L and 10% < 50 nmol/L).

**Figure 1:**
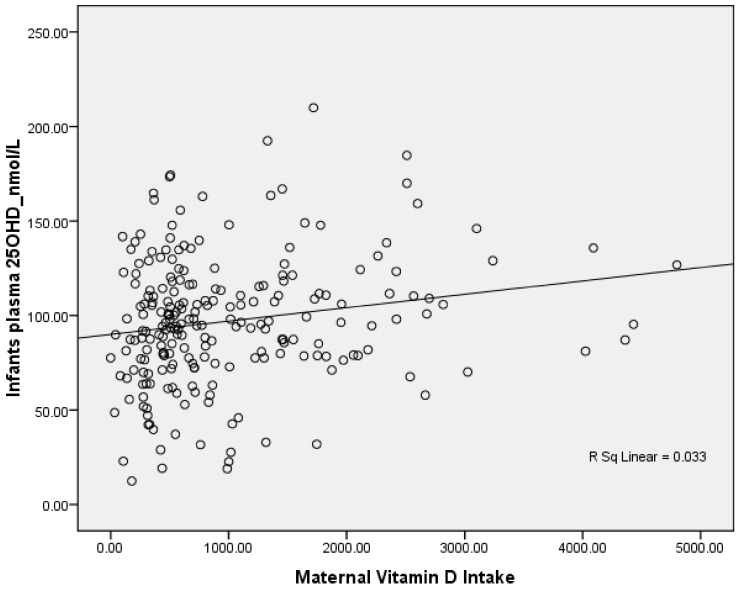
Correlation between infants’ plasma 25(OH)D and maternal vitamin D intake at 3 months postpartum.

Maternal mean age was 31.3 ± 4.5, and participants were primarily Caucasian (82%) with a pre-pregnancy body mass index (BMI) of 24.2 ± 4.7. Fifty-eight percent of participants were primiparous, 60% had a trade or university degree, and 60% had income higher than $100,000 CDN. The median estimated maternal vitamin D intake from diet, supplements, and both during the postpartum period were 184, 400, and 665 IU/day, respectively. Twenty-three percent of lactating mothers were not meeting the Estimated Average Requirements (EAR) for vitamin D (400 IU/day). There was a positive correlation between maternal daily dietary vitamin D intake and infants’ plasma 25(OH)D concentration in infants exclusively breastfed at three months of age (*r* = 0.18, *p* = 0.006) (Figure 1). In addition, there was a positive correlation between maternal daily dietary vitamin D intake and infants’ plasma 3-epi-25(OH)D_3_ concentration in infants exclusively breastfed at three months of age (*r* = 0.27, *p* = 0.01). However, there was no relationship between infants’ plasma 25(OH)D at three months of age and second trimester maternal plasma 25(OH)D (*r* = 0.07, *p* = 0.32).

There was a significant association between infants’ plasma 25(OH)D and maternal reported vitamin D intake at three months postpartum in an adjusted linear regression model (β = 0.008, 95% CI 0.002, 0.13). Infants’ plasma 25(OH)D was also associated with infants’ vitamin D intake (β = 16.31, 95% CI 2.69, 29.93) and birth weight (β = −0.013, 95% CI −0.023, −0.002). Season of sampling, race, and second trimester maternal plasma 25(OH)D were not significantly associated with infants’ plasma 25(OH)D concentration (Table 3). With each 100 IU increase in vitamin D intake by mothers, the infants’ total plasma 25(OH)D increased by 0.9 nmol/L when controlling for race, season, second trimester maternal plasma 25(OH)D, birth weight, and whether the infant received a daily vitamin D supplement.

## 4. Discussion

We found that one-fourth of exclusively breastfed infants had 25(OH)D concentration <75 nmol/L despite the fact that 90% of mothers reported providing a daily vitamin D supplement. Although we did not have data on the dose of vitamin D supplement in infants, the commercial Canadian vitamin D drop for infants contain 400 IU/drop [10], suggesting that this alone is not sufficient to ensure optimal status. Consistent with this, we showed that there is a significant relationship between maternal postpartum vitamin D intake from both diet and supplements and the vitamin D status of infants who were supplemented at three months of age. Although the IOM [11] current recommendation for insufficient vitamin D status is 25(OH)D <50 nmol/L, this is not considered optimal status by other organizations [17,18]; we therefore reported our findings based on 25(OH)D <75 nmol/L. Nevertheless, we found that 10% of exclusively breasted infants in our samples had concentrations of 25(OH)D <50 nmol/L despite receiving a daily vitamin D supplement.

Previous clinical trials have reported improved infant vitamin D status with either increasing maternal vitamin D intake [23,24,25] or infants’ vitamin D supplements [27]; however, to our knowledge, no study has reported the impact of both maternal vitamin D intake and infants receiving the current daily vitamin D recommended supplement on infant’s vitamin D status. Although previous studies have shown that breastfed infants who are not provided vitamin D supplements are reliant on their mothers’ vitamin D intake [23,24,25], our study showed that breastfed infants’ vitamin D status depends on their mother’s vitamin D intake even in those infants who are supplemented by vitamin D. Our analysis of breastfeeding mothers in the APrON study has shown that 23% were not meeting the EAR of 400 IU/day, which would negatively impact infants’ vitamin D status and health. These results suggest that, to ensure infant optimal vitamin D status, not only do infants require a supplement, but women also need to meet current vitamin D intake during breastfeeding, which for the majority of women would require a supplement. This study also provides evidence that the current IOM recommendation for breastfeeding mothers is not sufficient to ensure adequate vitamin D status of infants.

Our results contribute to the growing literature on vitamin D status and intake in breastfeeding mothers and infants worldwide [23,24,25,26,31,32,33,34,35]. Several recent studies have shown low vitamin D status is common in exclusively breastfed infants and their mothers and is associated with sun exposure and vitamin D supplementation [31,32,33,34,35]. A few RCTs have shown that maternal supplementation with vitamin D during the breastfeeding period is an effective way to increase infants’ vitamin D status in those not receiving supplementation. These studies have used different doses, from 2000 IU/day [20] to 6400 IU/day [25], or high doses of vitamin D (60,000 daily for 10 days), for better compliance [24]. Vitamin D status of exclusively breastfed infants depends on sunlight exposure, vitamin D store at birth, and secretion in breast milk [31,32,33,34,35]. A study of breastfeeding mothers from different geographic areas (latitude range 3° N to 54° N) has shown that the vitamin D content of breast milk is well below the IOM recommended dose of 400 IU/day for 0–6-month-old infants [33]. A review of 22 case reports (166 infants) with nutritional rickets during 1986–2003 from the United States showed that 96% of children were breastfed and only 5% of them reported receiving a vitamin D supplementation [36].

Vitamin D supplementation of all breastfed infants has been recommended by the IOM [11] and other organizations [10,37,38]. In our study, compliance rate of vitamin D supplementation in breastfeeding infants was high (90%); however, other studies have shown low compliance rates of vitamin D supplementation in breastfeeding infants from 2–19% [39,40,41,42]. A longitudinal study of effectiveness of supplementation of vitamin D in Japanese breastfed infants showed that adequate vitamin D intake is effective in preventing vitamin D deficiency in infants when their mothers actively participated in the study [31]. However, another US study was not able to make a valid assessment of the effect of infants’ supplementation on their status due to the low number of infants supplemented with vitamin D [35].

Our study had a lower rate of vitamin D insufficiency in infants and their mothers compared to other studies [13,14,15,16,31,32,33,34,35], which is due to the high compliance rate of taking vitamin D supplements by both mothers and their infants in the APrON cohort. In addition, a higher rate of mothers (72%) exclusively breasted their infants in the first 3–6 months of life, which is higher than the national average. In Canada, 89% of mothers breastfed their infant in 2011–2012, and 26% were breastfeeding exclusively for six months [43]. The high compliance rate of supplementation as well as breastfeeding in our study is likely due to the cohort being well educated and of high socio-economic status, which could influence the generalizability of study findings to the entire population. Another limitation of our study is the lack of information on sun exposure, skin color, and clothing coverage of participants, as well as the quantity of vitamin D supplementation in infants. However, due to the long winters in Alberta and Calgary’s high latitude (51° N), the effect of sun exposure and dermal synthesis may be less critical. In addition, given that vitamin D from food in Canada makes such a small overall contribution to vitamin D dietary intake, the major factor that could affect vitamin D status is supplement intake.

We also demonstrated the presence of 3-epi-25(OH)D_3_ in all infants’ plasma samples. Overall, the epimer contributed 15% of total vitamin D status, and it was very high in some infants (range 0.8–55.64). This finding is consistent with previous studies showing that epimer can alter vitamin D status estimation of pregnant women at the time of delivery and in their cord blood [29,44]. Previous studies have shown factors such as age, season, and vitamin D supplementation can affect 3-epi-25(OH)D_3_ [44]. In our study, there was a significant positive correlation between infants’ plasma 3-epi-25(OH)D_3_ and maternal vitamin D intake. In addition, 90% of infants were given supplements. This again suggests that vitamin D supplements could be a source for 3-epi-25(OH)D_3_, as shown in previous studies [29,44].

## 5. Conclusions

Our findings suggest that to ensure infant optimal vitamin D status, not only do infants require a supplement, but women also need to take supplemental intakes (>400 IU/day) of vitamin D during breastfeeding.

## Figures and Tables

**Table 1:** Characteristics of infants.

Characteristics	All Infants (*n* = 224)	Exclusively Breastfed Infant (*n* = 152)
Male, *n* (%)	122 (54.5)	83 (54.6)
Female, *n* (%) ^1^	102 (44.6)	69 (45.4)
Ethnicity (*n* = 224)	204 (91.1)	144 (94.7)
Caucasian, *n* (%)	204 (91.1)	144 (94.7)
Other, *n* (%)	20 (8.9)	8 (5.3)
Vitamin D intake ^2^, *n* (%)	172 (76)	139 (91)
25(OH)D (D_2_ + D_3_) (Median (25th–75th)) nmol/L	96.0 (77.6–116.2)	94.5 (76.9–115.1)
3-epi-25(OH)D_3_ nmol/L	15.6 ± 11.2	15.7 ± 10.9
Birth Weight (g) (Mean ± SD)	3377.8 ± 478.3 (*n* = 212)	3444.4 ± 444.9 (*n* = 142)
Birth Length (cm) (Mean ± SD)	50.9 ± 2.7 (*n* = 209)	51.2 ± 2.5 (*n* = 140)
Birth Head Circumference (cm) (Mean ± SD)	34.5 ± 1.6 (*n* = 211)	34.6 ± 1.5 (*n* = 142)

^1^ Gender is missing for two infants. ^2^ Infants’ vitamin D dose was not collected. SD: standard deviations.

**Table 2:** Vitamin D status (nmol/L) of infants and mothers.

**Infants (*n* = 224)**	**Mean ± SD**	**Median (25th to 75th)**	**Range (Min–Max)**
25(OH)D_3_	96.1 ± 33.0	95.5 (77.6–116.2)	197.4 (12.5–209.9)
25(OH)D_2_	0.1 ± 0.5	0	3.5 (0–3.5)
25(OH)D (D_2_ + D_3_)	97.1 ± 33.9	96.0 (77.6–116.2)	197.43 (12.5–209.9)
3-epi-25(OH)D_3_	15.6 ± 11.2	11.66 (7.7–21.2)	54.8 (0.8–55.6)
**Mothers (*n* = 224)**			
25(OH)D_3_	73.8 ± 28.6	75.5 (49.1–95.1)	145.3 (17.8–163.1)
25(OH)D_2_	2.2 ± 1.8	1.8 (0–3.4)	14.9 (0–14.9)
25(OH)D (D_2_ + D_3_)	76.0 ± 29.6	77.7 (49.1–95.1)	148.2 (17.8–166.0)
3-epi-25(OH)D_3_	4.3 ± 2.1	4.3 (2.5–5.7)	11.5 (0.4–11.9)

**Table 3:** Multiple regression model for 25(OH)D of infants.

Item	25(OH)D ^a^, β (95% CI)	*p*
Maternal vitamin D intake	0.008 (0.002, 0.13)	0.010
Infants’ vitamin D intake	16.31 (2.69, 29.93)	0.019
Season ^a^	−6.49 (−16.86, 3.88)	0.219
Birth weight	−0.013 (−0.23, −0.002)	0.024
Maternal 25(OH)D ^b^	0.50 (−0.12, 0.22)	0.564
Race ^c^	−2.93 (−20.01, 14.14)	0.735

^a^ Summer (3 May–31 October) versus winter (1 November–30 April). ^b^ nmol/L. ^c^ Caucasian versus non-Caucasian. CI: confidence interval.

## References

[1] Mahon P., Harvey N., Crozier S., Inskip H., Robinson S., Arden N., Swaminathan R., Cooper C., Godfrey K. (2010). SWS Study Group. Low maternal vitamin D status and fetal bone development: Cohort study. J. Bone Miner. Res..

[2] Weiler H., Fitzpatrick-Wong S., Veitch R., Kovacs H., Schellenberg J., McCloy U., Yuen C.K. (2005). Vitamin D deficiency and whole body and femur bone mass relative to weight in healthy newborns. Can. Med. Assoc. J..

[3] Viljakainen H.T., Saarnio E., Hytinantti T., Miettinen M., Surcel H., Mäkitie O., Andersson S., Laitinen K., Lamberg-Allardt C. (2010). Maternal vitamin D status determines bone variables in the newborn. J. Clin. Endocrinol. Metab..

[4] Aghajafari F., Nagulesapillai T., Ronksley P.E., Tough S.C., O’Beirne M., Rabi D. (2013). Association between maternal serum 25-hydroxyvitamin D level and pregnancy and neonatal outcomes: Systematic review and meta-analysis of observational studies. BMJ.

[5] Hyppönen E., Läärä E., Reunanen A., Järvelin M.R., Virtanen S.M. (2001). Intake of vitamin D and risk of type 1 diabetes: A birth-cohort study. Lancet.

[6] Mohr S.B., Garland C.F., Gorham E.D., Garland F.C. (2008). The association between ultraviolet B irradiance, vitamin D status and incidence rates of type 1 diabetes in 51 regions worldwide. Diabetologia.

[7] Hollis B.W., Wagner C.L. (2004). Vitamin D requirements during lactation: High-dose maternal supplementation during lactation as therapy to prevent hypovitaminosis D for both the mother and the nursing infant. Am. J. Clin. Nutr..

[8] Wagner C.L., Hulsey T.C., Fanning D., Ebeling M., Hollis B.W. (2006). High-dose vitamin D_3_ supplementation in a cohort of breastfeeding mothers and their infants: A 6-month follow-up pilot study. Breastfeed. Med..

[9] Widdowson E.M. (1971). Food intake and growth in the newly-born. Proc. Nutr. Soc..

[10] Health Canada Vitamin D and Calcium: Updated Dietary Reference Intakes. http://www.hc-sc.gc.ca/fn-an/nutrition/vitamin/vita-d-eng.php.

[11] Ross A.C., Taylor C.L., Yaktine A.L., Del Valle H.B., Institute of Medicine (US) Committee to Review Dietary Reference Intakes for Calcium and Vitamin D (2011). Dietary Reference Intakes for Calcium and Vitamin D.

[12] Aghajafari F., Field C.J., Kaplan B.J., Rabi D., Maggiore J.A., O’Beirne M., Hanley D.A., Eliasziw M., Dewey D., Weinberg A. (2016). The current recommended vitamin D intake guideline for diet and supplements during pregnancy is not adequate to achieve vitamin D sufficiency for most pregnant women. PLoS ONE.

[13] Kramer C.K., Ye C., Swaminathan B., Hanley A.J., Connelley P.W., Sermer M., Zinman B., Retnakaran R. (2016). The persistence of maternal vitamin D deficiency and insufficiency during pregnancy and lactation irrespective of season and supplementation. Clin. Endocrinol..

[14] Wangyang L., Green T.J., Innis S.M., Barr S.I., Whiting S.J., Shand A., von Dadelszen P. (2011). Suboptimal vitamin D levels in pregnant women despite supplemental use. Can. J. Public Health.

[15] Gellert S., Strohle A., Hahn A. (2017). Breastfeeding women are at higher risk of vitamin D deficiency that non-breastfeeding women—Insights from the German VitaMinFemin study. Int. Breastfeed. J..

[16] Wheeler B.J., Taylor B.J., de Lange M., Harper M.J., Jones S., Mekhail A., Houghton L.A. (2018). A longitudinal study of 25-hydroxy Vitamin D and parathyroid hormone status throughout pregnancy and exclusive lactation in New Zealand mothers and their infants at 45° S. Nutrients.

[17] Hanley D.A., Cranney A., Jones G., Whiting S.J., Leslie W.D., Cole D.E.C., Atkinson S.A., Josse R.G., Feldman S., Kline G.A. (2010). Vitamin D in adult health and disease: A review and guideline statement from Osteoporosis Canada. Can. Med. Assoc. J..

[18] Holick M.F., Binkley N.C., Bischoff-Ferrari H.A., Gordon C.M., Hanley D.A., Heaney R.P., Murad M.H., Weaver C.M., Endocrine Society (2011). Evaluation, treatment, and prevention of vitamin D deficiency: An Endocrine Society clinical practice guideline. J. Clin. Endocrinol. Metab..

[19] Bischoff-Ferrari H.A., Dietrich T., Orav E.J., Hu F.B., Zhang Y., Karlson E.W., Dawson-Hughes B. (2004). Higher 25-hydroxyvitamin D concentrations are associated with better lower-extremity function in both active and inactive persons aged ≥60 years. Am. J. Clin. Nutr..

[20] Heaney R.P. (2004). Functional indices of vitamin D status and ramifications of vitamin D deficiency. Am. J. Clin. Nutr..

[21] Bischoff-Ferrari H.A., Willett W.C., Wong J.B., Giovannucci E., Dietrich T., Dawson-Hughes B. (2005). Fracture prevention with vitamin D supplementation: A meta-analysis of randomized controlled trials. JAMA.

[22] Gaugris S., Heaney R.P., Boonen S., Kurth H., Bentkover J.D., Sen S.S. (2005). Vitamin D inadequacy among post-menopausal women: A systematic review. QJM.

[23] Ala-Houhala M., Koskinen T., Parviainen M.T., Visakorpi J.K. (1988). 25-25-hydroxyvitamin D and vitamin D in human milk: Effects of supplementation and season. Am. J. Clin. Nutr..

[24] Naik P., Faridi M.M.A., Batra P., Madhu S.V. (2017). Oral supplementation of parturient mothers with vitamin D and its effect on 25OHD status of exclusively breastfed infants at 6 months of age: A double-blind randomized placebo controlled trial. Breastfeed. Med..

[25] Hollis B.W., Wagner C.L., Howard C.R., Ebeling M., Shary J.R., Smith P.G., Taylor S.N., Morella K., Lawrence R.A., Hulsey T.C. (2015). Maternal versus infant vitamin supplementation during lactation: A randomized controlled trial. Pediatrics.

[26] Galo S., Comeau K., Vanstone C., Agellon S., Sharma A., Jones G., L’Abbé M., Khamessan A., Rodd C., Weiler H. (2013). Effect of different dosages of oral vitamin D supplementation on vitamin D status in healthy, breastfed infants: A randomized trial. JAMA.

[27] Kaplan B.J., Giesbrecht G.F., Leung B.M.Y., Field C.J., Dewey D., Bell R.C., Manca D.P., O’Beirne M., Johnston D.W., Pop V.J. (2014). The Alberta Outcomes and Nutrition (APrON) cohort study: Rationale and methods. Matern. Child Nutr..

[28] Gomez F.M., Field C.J., Olstand D.L., Loehr S., Ramage S., McCargar L.J., APrON Study Team (2015). Use of micronutrient supplements among pregnant women in Alberta: Results from the Alberta Pregnancy Outcomes and Nutrition (APrON) cohort. Matern. Child Nutr..

[29] Aghajafari F., Field C.J., Rabi D., Kaplan B.J., Maggiore J.A., O’Beirne M., Hanley D.A., Eliasziw M., Dewey D., Ross S.J. (2016). Plasma 3-epi-25-hydroxycholecalciferol can alter the assessment of vitamin D status using the current reference ranges for pregnant women and their newborns. J. Nutr..

[30] Mahsa J., Farmer A.P., Maximova K., Willows N.D., Bell R.C., APrON Study Team (2013). Predictors of exclusive breastfeeding: Observations from the Alberta pregnancy outcomes and nutrition (APrON) study. BMC Pediatr..

[31] Terashita S., Nakamura T., Igarashi N. (2017). Longitudinal study on the effectiveness of vitamin D supplements in exclusively breast-fed infants. Clin. Peditr. Ednocrinol..

[32] Dawodu A., Davisson B., Woo J., Peng Y.-M., Ruiz-Palacios G.M., de Lourdes Guerrero M., Morrow A.L. (2015). Sun exposure and vitamin D supplementation in relation to vitamin D status of breastfeeding mothers and infants in the global exploration of human milk study. Nutrients.

[33] Stoutjesdijk E., Schaafsma A., Nhien N.V., Lin Khor G., Kema I.P., Janneke Dijck-Brouwer D.A., Muskiet F.A.J. (2017). Milk vitamin D in relation to the ‘adequate intake’ for 0–6-month-old infants: A study in lactation women with different cultural backgrounds, living at different latitudes. Br. J. Nutr..

[34] Darmawikarta D., Chen Y., Lebovic G., Birken C.S., Parkin P.C., Maguire J.L. (2016). Total duration of breastfeeding, vitamin D supplementation, and serum levels of 25-hydroxyvitamin D. Am. J. Public Health.

[35] Dawodu A., Zalla L., Woo J.G., Herbers P.M., Davidson B.S., Heubi J.E., Morrow A.L. (2014). Heightened attention to supplementation is needed to improve the vitamin D status of breastfeeding mothers and infants when sunshine exposure is restricted. Matern. Child Nutr..

[36] Weisberg P., Scanlon K.S., Li R., Cogswell M.E. (2004). Nutritional rickets among children in the United States: Review of cases reported between 1986 and 2003. Am. J. Clin. Nutr..

[37] Gartner L.M., Greer F.R., Section on Breastfeeding and Committee on Nutrition, American Academy of Pediatrics (2003). Prevention of rickets and vitamin D deficiency: New guidelines for vitamin D intake. Pediatrics.

[38] Godel J.C., Canadian Pediatrics Society, First Nations, Inuit and Métis Health Committee (2007). Vitamin D supplementation: Recommendation for Canadian mothers and infants. Pediatr. Child Health.

[39] Taylor J.A., Geyer L.J., Feldman K.W. (2010). Use of supplemental vitamin D among infants breastfed for prolonged periods. Pediatrics.

[40] Gordon C.M., Feldman H.A., Sinclair L., LeBoff Williams A., Kleinman P.K., Perez-Rossello J., Cox J.E. (2008). Prevalence of vitamin D deficiency among healthy infants and toddlers. Arch. Pediatr. Adolesc. Med..

[41] Perrine C.G., Sharma A.J., Jefferds M.E., Serdula M.K., Scanlon K.S. (2010). Adherence to vitamin D recommendations among US infants. Pediatrics.

[42] Ahrens K.A., Rossen L.M., Simon A.E. (2015). Adherence to vitamin D recommendations among U.S. infants aged 0 to 11 months, NHANES, 2009 to 2012. Clin. Pediatr..

[43] Gonet L., Health at a Glance Breastfeeding Trends in Canada. https://www.statcan.gc.ca/pub/82-624-x/2013001/article/11879-eng.htm.

[44] Bailey D., Veljkovic K., Yazdanpanah M., Adeli K. (2013). Analytical measurement and clinical relevance of vitamin D (3) C3-epimer. Clin. Biochem..

